# Hepcidin antagonists for potential treatments of disorders with hepcidin excess

**DOI:** 10.3389/fphar.2014.00086

**Published:** 2014-04-28

**Authors:** Maura Poli, Michela Asperti, Paola Ruzzenenti, Maria Regoni, Paolo Arosio

**Affiliations:** Molecular Biology Laboratory, Department of Molecular and Translational Medicine, University of BresciaBrescia, Italy

**Keywords:** hepcidin, heparin, anemia of chronic diseases, inflammation, iron metabolism

## Abstract

The discovery of hepcidin clarified the basic mechanism of the control of systemic iron homeostasis. Hepcidin is mainly produced by the liver as a propeptide and processed by furin into the mature active peptide. Hepcidin binds ferroportin, the only cellular iron exporter, causing the internalization and degradation of both. Thus hepcidin blocks iron export from the key cells for dietary iron absorption (enterocytes), recycling of hemoglobin iron (the macrophages) and the release of storage iron from hepatocytes, resulting in the reduction of systemic iron availability. The BMP/HJV/SMAD pathway is the major regulator of hepcidin expression that responds to iron status. Also inflammation stimulates hepcidin via the IL6/STAT3 pathway with a support of an active BMP/HJV/SMAD pathway. In some pathological conditions hepcidin level is inadequately elevated and reduces iron availability in the body, resulting in anemia. These conditions occur in the genetic iron refractory iron deficiency anemia and the common anemia of chronic disease (ACD) or anemia of inflammation. Currently, there is no definite treatment for ACD. Erythropoiesis-stimulating agents and intravenous iron have been proposed in some cases but they are scarcely effective and may have adverse effects. Alternative approaches aimed to a pharmacological control of hepcidin expression have been attempted, targeting different regulatory steps. They include hepcidin sequestering agents (antibodies, anticalins, and aptamers), inhibitors of BMP/SMAD or of IL6/STAT3 pathway or of hepcidin transduction (siRNA/shRNA) or ferroportin stabilizers. In this review we summarized the biochemical interactions of the proteins involved in the BMP/HJV/SMAD pathway and its natural inhibitors, the murine and rat models with high hepcidin levels currently available and finally the progresses in the development of hepcidin antagonists, with particular attention to the role of heparins and heparin sulfate proteoglycans in hepcidin expression and modulation of the BMP6/SMAD pathway.

## HEPCIDIN DISCOVERY AND PROPERTIES

Hepcidin was independently discovered in the years 2000–2001 by various groups. Krause et al. (2000) isolated from human blood ultrafiltrate a 25-residue peptide with antimicrobial activity that they named LEAP-1 (liver-expressed antimicrobial peptide 1). In the same period Pigeon et al. (2001) identified an iron-regulated gene that encoded for the LEAP-1 with high expression in the liver, and much lower expression in the kidney, adipose tissue, heart, and brain. Park et al. (2001) characterized a cysteine-rich peptide and named it hepcidin (hepatic bactericidal protein) for its hepatic origin with a structure typical for an antimicrobial activity. They found homologous cDNAs in the liver of various species from fish to human. A central role of hepcidin in systemic iron homeostasis was soon unambiguously recognized by the finding that inactivation of its gene was associated with severe iron overload in the liver and pancreas (Nicolas et al., 2001). The finding was serendipitous, since the knockout construct was aimed at deleting USF2 gene (upstream stimulatory factor 2) but it also removed the hepcidin adjacent genes. A following specific USF2 knockout mouse had normal hepcidin and iron, while the specific inactivation of hepcidin gene caused iron overload (Nicolas et al., 2002). The importance of hepcidin was conclusively demonstrated by the finding that transgenic mice overexpressing the peptide showed a severe and often lethal anemia (Nicolas et al., 2003). The initial excitement about hepcidin as a central player in the communication of body iron stores to the intestinal absorptive cells (Fleming and Sly, 2001) was further sustained by the finding that patients with homozygous mutations in the hepcidin gene were affected by severe juvenile hemochromatosis (Roetto et al., 2003). The following years were dedicated to the characterization of the gene, its product and the regulation of its expression.

**FIGURE 1:**
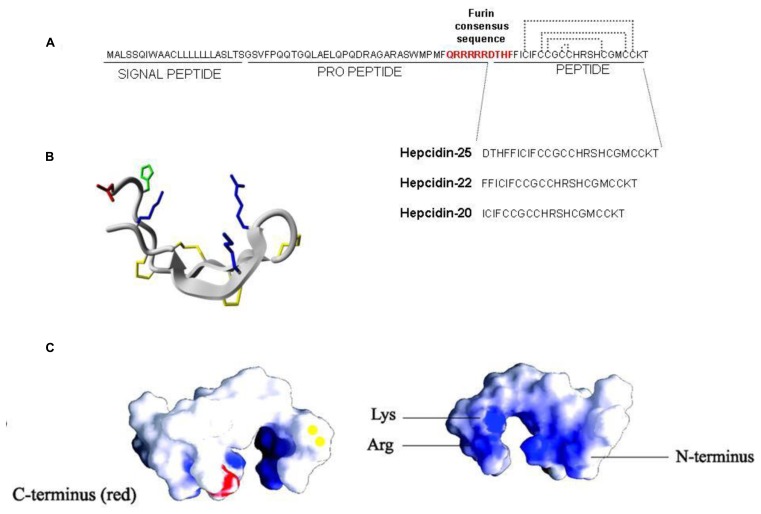
**Hepcidin structure. (A)** Hepcidin is encoded as a precursor prepropeptide of 84 amino acids, it is processed by two sequential cleavages. The first cleavage is for removing the signal peptide and the second the pro-peptide to produce the mature peptide of 25 amino acids. Furin is the convertase that recognizes the consensus sequence QRRRRRDTHF forming three different hepcidin (25; 22 and 20). **(B)** The structure of hepcidin-25 is characterized by two short β-strands stabilized by four interstrand disulfide bonds. **(C)** The view of space-filling diagram of hepcidin-25 indicating cationic charges (blue; the right figure) and hydrophobic (white) side with vicinal disulfide (yellow) and the C-terminus in red (the left figure). Structures were generated with GRASP and derived from Hunter et al. (2002).

In human the hepcidin gene is in chromosome 19 and encodes a precursor prepropeptide of 84 amino acids that is processed by two sequential cleavages. The first of the signal sequence and the second of the pro-region to produce the mature peptide of 25 amino acids (aa). Furin, a major member of the family of prohormone convertases, is the enzyme involved in the processing, and it recognizes the consensus sequence (QRRRRR↓DTHF) conserved in mammal and fish hepcidins (Shike et al., 2004; Valore and Ganz, 2008; **Figure 1A**). Chemical or siRNA-mediated inhibition of furin prevents hepcidin maturation but not its secretion from the cell (Valore and Ganz, 2008). The processing may be more complex, since two additional hepcidin N-terminal truncated forms of 22 and 20 aa were originally described (**Figure 1A**). Hepc-25 and hepc-20 are formed intracellularly and possibly processed in the Golgi apparatus by furin-like proteases and both secreted in the blood. Whereas hepc-22 is present only in urine (Park et al., 2001). The role of hepc-20 and hepc-22 is not clear. The NMR structure of the mature hepcidin 25 was obtained from the refolded synthetic peptide (Jordan et al., 2009). It consists of two short β-strands stabilized by interstrand disulfide bonds (**Figure 1B**). It has amphipathic properties with cationic charges and hydrophobic surface like most of antimicrobial peptides (**Figure 1C**), however, the antimicrobial activity of human hepcidin is low and probably not critical. Mouse has two different hepcidin genes (hepc-1 and hepc-2) but only hepc-1 is related with iron metabolism.

Although mammalian hepcidin attracted most interest, it should be mentioned that hepcidin has been described also in fishes, where the size and cysteines are conserved, but the overall amino acid sequence identity with the human one is only 50% (Nemeth and Ganz, 2006). The role of fish hepcidin in systemic iron regulation is unclear.

## REGULATION OF HEPCIDIN EXPRESSION IN THE LIVER

**FIGURE 2:**
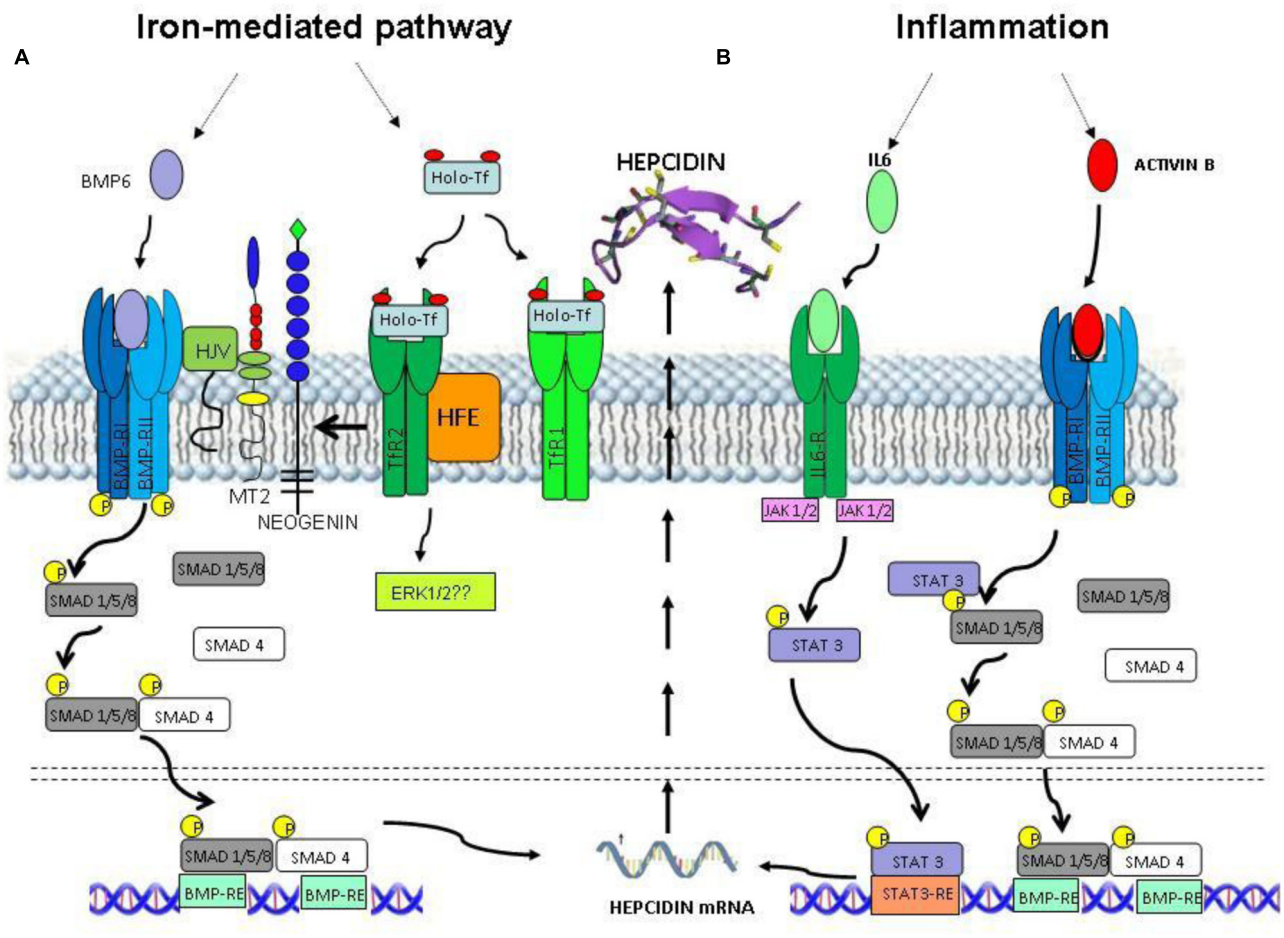
**Intracellular signaling pathways for hepcidin expression. (A)** Iron status stimulates hepcidin expression through BMP6 and Holo-Tf. This signaling involves BMP receptor, HJV (BMPco-receptor), TMPRSS6 (MT2, a negative regulator), Neogenin, TfR2, and HFE (sensors of Holo-Tf saturation). Intracellularly this multiprotein complex triggers the activation of SMAD1/5/8 and SMAD4 components and possibly ERK1/2. **(B)** The inflammatory status triggers the hepcidin expression through IL6/STAT3 pathway and ActivinB that activates SMAD1/5/8 pathway.

Most of the regulation of hepcidin expression in the liver occurs at transcriptional level and it is modulated by iron status, inflammation, and hypoxia (Nemeth and Ganz, 2006). Iron supplementation to the animal readily stimulates liver hepcidin mRNA, but this does not occur in cultured cells, of hepatic or non-hepatic lineage, suggesting an indirect mechanism of iron sensing. A breakthrough for the understanding of the regulatory mechanism was the finding that mice with liver conditional inactivation of the SMAD4 gene developed early and severe liver iron overload, since they did not express detectable hepcidin mRNA even after induction with iron and inflammatory stimuli (Wang et al., 2005). SMAD4 is the key player of the TGF-beta/BMP signaling transduction that involves serine kinases, and phosphorylation of SMAD members to form complexes with SMAD4. These complexes enter the nucleus and bind the responsive elements of the target genes for their activation. The centrality of this regulatory pathway was confirmed by the finding that hemojuvelin (HJV), which is mutated in severe juvenile hemochromatosis (Papanikolaou et al., 2004), acts as a co-receptor of the BMP/SMAD pathway in the liver (Babitt et al., 2006). The 20 different members of the BMP family are produced by various cell types and have different functions, including osteogenesis, embryogenesis, and differentiation. Initial experiments used the osteogenic BMP2 and BMP4 to stimulate hepcidin expression in cultured hepatic cells (Truksa et al., 2006). *In vitro* studies showed that also BMP5, 7 and 9 can induce SMAD pathway and hepcidin expression in primary hepatocytes (Truksa et al., 2006) but after the finding that BMP6 is modulated by systemic iron and, more important, that BMP6^-^^/^^-^ mice suffer of severe iron overload and the lack of liver hepcidin it was accepted that BMP6 is the major regulator of hepcidin expression (Andriopoulos et al., 2009; Meynard et al., 2009). The dimers of type-II and type-I BMP-receptor participate in BMP/SMAD signaling together with various co-receptors and inhibitors. In the hepatic signaling, ALK2/ALK3 are the predominant BMPR type-I, and ActRIIA is the predominant type-II (Xia et al., 2008) and, of note, the GPI-anchor protein HJV acts as an essential co-receptor for hepcidin expression (Babitt et al., 2006). HJV is a member of the repulsive guidance molecule (RGM) family, which includes RGMa and DRAGON (RGMb), GPI-anchored proteins apparently involved in BMP signaling in different tissues (Corradini et al., 2009). HJV is expressed in skeletal and heart muscle and particularly in the liver where acts as an essential regulator of the signaling. It is also processed by the convertase furin into a soluble form that may act as a decoy and reduce hepcidin expression (Kuninger et al., 2008; Silvestri et al., 2008). It is degraded by the liver-specific serine protease Matriptase-2 (MT2, alias *TMPRSS6*; Silvestri et al., 2009). Inactivation of MT2 in mice and in human results in hepcidin increase and causes iron refractory iron deficiency anemia (IRIDA; Du et al., 2008; Finberg et al., 2008; Folgueras et al., 2008). Neogenin is another transmembrane protein involved in the signaling. It is ubiquitously expressed and interacts with RGM proteins. In the liver it interacts with both HJV and MT2 and may be required for the proper assembly of HJV-BMP ligand-BMPR-I/BMPR-II complex to initiate the BMP signaling and induce hepcidin expression (Zhang et al., 2005; Enns et al., 2012). Neogenin may also form a ternary complex with MT2 and HJV that facilitates HJV cleavage by MT2. Thus Neogenin can stimulate or suppress the BMP signaling by favoring the assembly of BMPs/HJV/BMPR complex or by facilitating MT2 activity, respectively (Zhao et al., 2013). A point not yet fully elucidated regards the involvement of HFE and TFR2. These genes are mutated in type I and III hemochromatosis, both characterized by relatively low hepcidin levels. HFE can bind both TFR1 and TFR2 and a model has been proposed in which HFE and TFR2 act as a complex and contribute to the complete activation of BMP/SMAD pathway (Gao et al., 2010). Other studies indicated that HFE and TFR2 are involved in the activation of MAPK, a pathway that cross talks with the BMP/SMAD and contribute to hepcidin expression (Poli et al., 2010; **Figure 2A**).

Regulation of the BMP/SMAD signaling occurs also after the phosphorylation of SMAD1/5/8 and ensures fine tuning at cytosolic level. SMAD7 antagonizes the recruitment of SMAD4 and the translocation of SMAD1/5/8-SMAD4 complex into the nucleus and recently it has been demonstrated that in hepcidin promoter there is a SMAD7-binding motif for a direct inhibition of the promoter (Mleczko-Sanecka et al., 2010).

Another well characterized pathway of hepcidin regulation involves the inflammatory cytokine IL6 that binds its specific receptor and activates JAK1/2 to phosphorylate STAT3. The phosphoSTAT3 translocates into the nucleus and binds the STAT3 response element (STAT3-RE) in hepcidin promoter, stimulating its transcription (Verga Falzacappa et al., 2007). This pathway can be activated by other cytokines including IL22 and Oncostatin-M which also increase hepcidin transcription, (Chung et al., 2010; Armitage et al., 2011). This inflammatory signaling relies on the BMPs/SMAD pathway to trigger hepcidin expression; in fact SMAD4-KO mice do not respond to the stimulation of IL6 (Wang et al., 2005). Activin-B, which belongs to the TGF-beta family, was shown to be stimulated by inflammation and to induce hepcidin via the BMP/SMAD pathway, thus it may cooperate and enhance the IL6-dependent stimulation and it could represent a connecting component between iron status and inflammation (Besson-Fournier et al., 2012; **Figure 2B**).

## THE HEPCIDIN–FERROPORTIN AXIS

**FIGURE 3:**
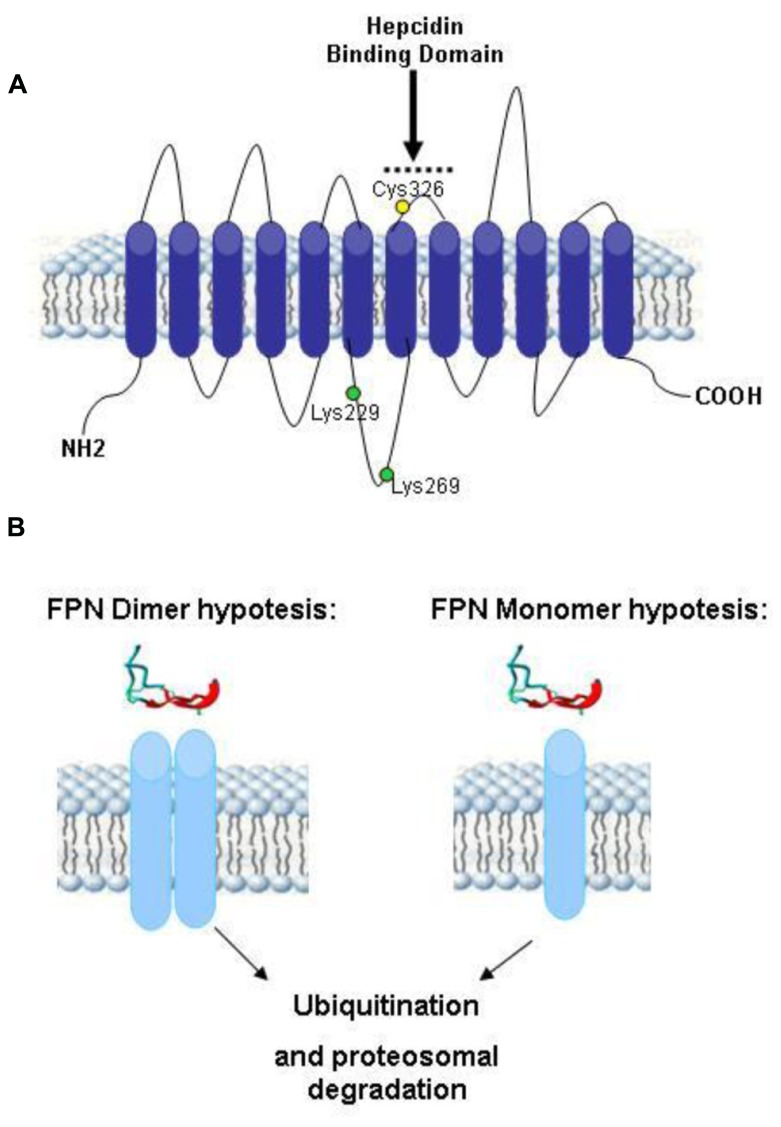
**Theoretical model of human ferroportin. (A)** A 2-dimensional model of ferroportin, based on the model of (Liu et al., 2005; Wallace et al., 2010), modified from Wallace et al. (2010). It shows a 12-transmembrane (TM) domain structure with both N- and C-terminus cytosolic. In yellow is marked Cys326 fundamental for hepcidin–ferroportin binding and in green Lys229 and 269 essential for ferroportin ubiquitination. **(B)** Theoretical models of ferroportin dimer and monomer after the binding of hepcidin. Both of them include the proteasoma degradation of ubiquitinated ferroportin.

The only known receptor for hepcidin is ferroportin, which is the sole cellular iron exporter. Ferroportin (FPN) is expressed by enterocytes of the duodenum, by macrophages that process effete RBC and by liver, and is responsible for the release of iron to transferrin, in a mechanism that needs the assistance of a copper ferroxidase such ceruloplasmin or haephestin (Nemeth et al., 2004; Hentze et al., 2010). The iron export activity of ferroportin is essential for the regulation of systemic iron homeostasis, but its biochemical properties and the mechanism of iron transport are not well characterized. It is a 62.5 kDa protein with 12 transmembrane domains, the N-terminus and possibly also the C-terminus are cytosolic (Liu et al., 2005; **Figure 3A**). Mutation in FPN gene lead to type IV hemochromatosis (ferroportin disease), which is dominantly transmitted. This has suggested that ferroportin forms functional dimers (De Domenico et al., 2005), but direct studies indicated that it is a monomer (Montosi et al., 2001; Liu et al., 2005; Wallace et al., 2010; **Figure 3B**). The ferroportin mRNA has an IRE on the 5′UTR that may be responsible for its iron-dependent regulation (Muckenthaler, 2008). However, the major regulatory step occurs at a post-translational level caused by hepcidin (Nemeth et al., 2004). In fact the binding of hepcidin causes ferroportin internalization and degradation, with the effect to reduce systemic iron availability (Ganz and Nemeth, 2012). Further studies showed that Hepcidin-25 binds an extracellular loop of FPN adjacent to the cytosolic loop containing the two tyrosines required to signal internalization (Nemeth et al., 2006). The binding involves disulfide bridging with FPN Cys326 (Fernandes et al., 2009; **Figure 3A**), ferroportin ubiquitination prevalently at the level of lysines present in a third cytoplasmic loop of ferroportin (possibly Lys 229 and 269), and proteasomal degradation (Qiao et al., 2012; **Figure 3B**).

## LOCAL EXPRESSION OF HEPCIDIN IN CNS

**FIGURE 4:**
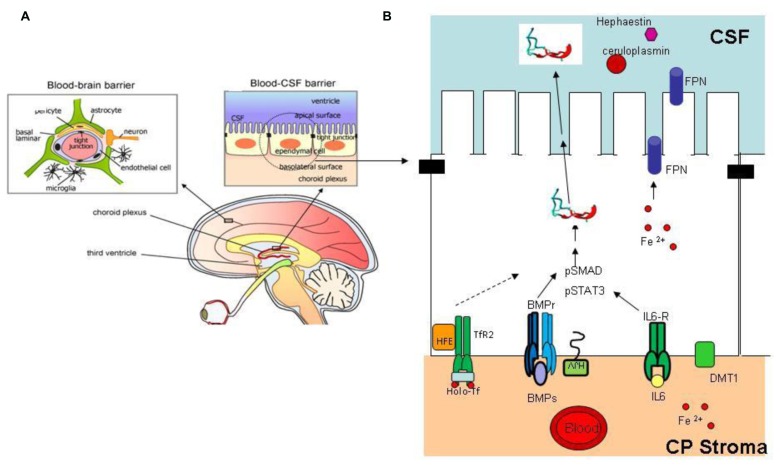
**Brain iron regulation. (A)** The blood–brain barrier and blood–CSF barrier are the two major sites of iron exchange with the periphery (Edelhauser et al., 2010). They act as a diffusion barrier to large and hydrophilic molecules. Small, lipophilic molecules or toxic metabolites are delivered through specific carrier mediating influx or efflux. The structure responsible for the correct working of these barriers is the tight junction (black rectangle); transferrin and its receptor are responsible for most of the iron uptake into CNS [modified from Edelhauser et al. (2010)]. **(B)** Schematic representation of an ependymal cell in the blood–CSF barrier. This cell type, like other cell types of the CNS, express proteins involved in hepcidin expression and iron regulation, ferroportin (for export to CSF), TfR2, HFE, BMPr, HJV, IL6r, DMT1. Inflammatory stimuli, iron deregulation, or other effectors that alter hepcidin expression may contribute to modify brain iron homeostasis and compartmentalization. Reprinted with permission from Choi and Kim (2008).

Liver is the major producer of hepcidin, and the fine tuning of its expression sets the level of circulating hepcidin to govern systemic iron homeostasis. Various other tissues express hepcidin mRNA and protein suggesting the presence of autocrine or paracrine circuits that may contribute to the regulation of local iron distribution. This may be particularly important in the central nervous system (CNS) that is separated from the rest of the body. The blood–brain barrier (BBB) and the blood/CSF barrier are the major sites of iron exchange with the periphery (Rouault et al., 2009; **Figure 4A**). These barriers act as semipermeable cellular gates characterized by tight junction and specialized transcellular carriers mediating influx or efflux. Transferrin and its receptor take part in most of iron transfer, but the details on the regulatory mechanism are missing. Of interest is that cells of the blood/CSF barrier express proteins of the hepcidin/ferroportin axis (**Figure 4B**). The imaging techniques and histology of the different areas of the brain showed that regions with neurodegeneration exhibit also iron accumulation in pathological conditions such as Alzheimer’s disease (AD; Bishop et al., 2002), Parkinson’s disease (PD; Berg et al., 2001; Zecca et al., 2004), and neurodegeneration with brain iron accumulation (NBIA; Hayflick, 2006), while restless leg syndrome (RLS) is associated with reduced CNS iron content (Clardy et al., 2006). There is evidence that the hepcidin/ferroportin axis might play a role in the iron decompartmentalization occurring in these disorders. In fact, not only hepcidin and ferroportin, but also the accessory proteins in iron transfer ceruloplasmin, hephaestin and DMT1, and those involved in hepcidin regulation (BMPs, IL6, Tfr2) are expressed and regulated in the brain (**Figure 4B**). Hepcidin and ferroportin mRNA and protein were detected in different murine and human cerebral areas (Clardy et al., 2006; Zechel et al., 2006) and hepcidin intraventricular injection resulted in downregulation of FPN protein (Wang et al., 2010b). Systemic iron inflammation or acute iron overload induced a significant increase in cerebral hepcidin expression in mice and rats (Malik et al., 2011; Wang et al., 2011; Sun et al., 2012) thus suggesting that the machinery controlling hepcidin expression in the brain is similar to the hepatic one. Novel evidence suggests that perturbation of the cerebral hepcidin-FPN axis may contribute to local deregulation of iron homeostasis. They include an increase of hepcidin in the brain in a murine model of cerebral ischemia (Ding et al., 2011), and a reduction of both ferroportin and hepcidin detected in lysates obtained from AD patients (Raha et al., 2013). Furthermore, calorie restriction prevented both the increase in cerebral hepcidin mRNA and the impairment of learning and memory observed in an experimental model of aging (Wei et al., 2014). Further characterization of the machinery controlling iron balance in the brain is needed, but attention should be given at the possible modulation of hepcidin expression in CNS caused by pharmacological intervention aimed at regulating systemic iron homeostasis, in particular those involving molecules that can pass the BBB.

## IRON DISORDERS IN WHICH HEPCIDIN IS PATHOLOGICALLY UPREGULATED

Hepcidin is the hormone of iron and inflammation and its deregulation occurs in all iron related disorders, including the ones characterized by iron restriction and anemia in which hepcidin is abnormal (Ganz and Nemeth, 2011). Elevated hepcidin levels are associated with secondary iron overload, genetic IRIDA, chronic infectious and inflammatory diseases resulting in anemia of inflammation (AI). The finding that hepcidin is upregulated by the inflammatory cytokine IL6 (Verga Falzacappa et al., 2007) contributed to explain the anemia of chronic diseases (ACD) alias AI. This occurs in a variety of disorders, like infections, chronic kidney diseases (CKDs), rheumatoid arthritis, and cancer (Wang et al., 2012) including multiple myeloma (Maes et al., 2010), a severe malignant disorder of plasma cells. Currently ACD therapy includes erythropoiesis-stimulating agents and intravenous iron (Goodnough et al., 2010), which may have adverse effects and are scarcely effective (Glaspy, 2012; Fung and Nemeth, 2013). For example inflammation often induces erythropoietin resistance (Macdougall and Cooper, 2002). Alternative treatments have been proposed and hepcidin antagonists seem to be the best candidates to treat these disorders (Wang et al., 2012).

## THERAPEUTIC APPROACHES TO NEUTRALIZE HEPCIDIN EXCESS

The mechanisms involved in the regulation of hepcidin expression are complex and partially known, and the approaches can use different targets to downregulate hepcidin or its function, as described in recent reviews (Sun et al., 2011; Fung and Nemeth, 2013).

### BMP/BMPR COMPLEX

One obvious target is the BMPs/BMPR complex. This was initially tested by developing anti-BMP6 antibodies to abolish the interaction between BMP6 and its receptors. The iron-restricted anemia of HFE transgenic mice due to high hepcidin was effectively cured with 10-day treatment with anti-BMP6 (Corradini et al., 2010). However, similar treatment was not effective in ACD possibly due to the expression of other BMPs, as it occurs in multiple myeloma with high BMP2 (Maes et al., 2010). Another target is HJV, the major co-receptor of the BMP/SMAD signaling. A treatment of healthy rodents with soluble HJV.Fc blocked SMAD phosphorylation, decreased hepcidin expression, mobilized splenic iron content and increased serum iron levels (Babitt et al., 2007). In an ACD rat model, a long 4-week treatment showed a recovery of anemia with the inhibition of SMAD1/5/8 phosphorylation; increase of splenic ferroportin levels and of serum iron (Theurl et al., 2011). Another promising approach is to block the phosphorylation of type I BMP receptor. The initially tested molecule was dorsomorphin, a non-specific kinase inhibitor (Yu et al., 2008), and then a more selective BMP inhibitor, coded LDN-193189 (Cuny et al., 2008), in animal models. A 4-week treatment of anemic rats with chemically induced arthritis reduced hepatic hepcidin mRNA levels, increased serum iron concentration, increased ferroportin expression in splenic macrophages, and improved hemoglobin levels and hematocrit (Theurl et al., 2011). It was also effective in treating mice with acute inflammatory anemia induced by turpentine injections (Steinbicker et al., 2011). A limit of this chemical inhibition of hepcidin is the lack specificity for BMP inhibition, since it can also potently inhibit VEGF and components of the MAPK/ERK pathway and show toxicity (Vogt et al., 2011). Liver is the organ most easily targeted by siRNAs, and the studies on the silencing of HJV and TfR2 are ongoing (Akinc et al., 2011).

### HEPARIN

**FIGURE 5:**
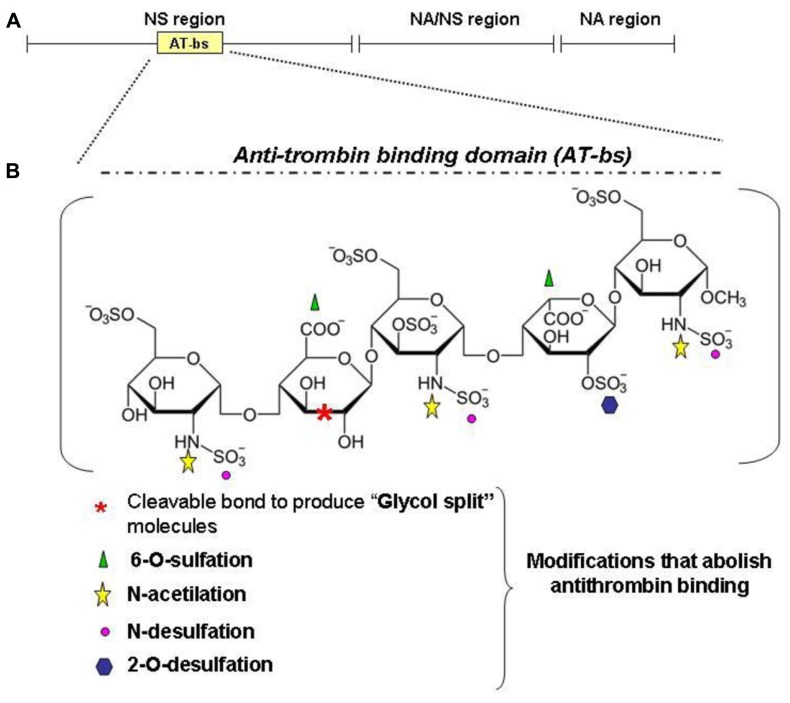
**Heparin structure. Heparin is a sulfated polysaccaride belonging to the glycosaminoglycans family.** It is composed of disaccharide units formed by one uronic acid (L-iduronic acid, IdoA, or D-glucuronic acid, GlcA) and one amino sugar (D-glucosamine, GlcN). IdoA is prevalently sulfated at the position 2 and GlcN is prevalently *N*-sulfated (or *N*-acetylated) and 6-*O*-sulfated. The main structure of heparin is composed by 70% of *N*-sulfated region (NS, IdoA2SO3 -GlcNSO36SO3), *N*-acetylated region (NA, GlcA-GlcNAc) and mixed NA/NS (GlcA-GlcNSO3; **A**). **(B)** An important region for the anticoagulant property of heparins is the antitrombin-binding domain (AT-bs), present in no more than one third of the chain, characterized by the pentasaccharidic sequence: GclNAc6SO3–GlcA-GlcNSO3-6SO3–IdoA2SO3-GlcNSO3.6SO3. Non-anticoagulant heparins can be obtained by removing the AT-bs or by modifying one or more residues essential for the binding to AT. For example: *N*-desulfation and 2-*O* or 6-*O*-desulfation cause a dramatic drop in the anticoagulant activity as *N*-acetylation. Other possibilities include the reduction of carboxyl groups of GlcA residue and the cleavage of the bond between its two hydroxyl groups to obtain the glycol-split heparins.

The observation that BMPs are heparin binding molecules and that heparin modifies the osteogenic activity of BMP2/4 stimulated (Poli et al., 2011) to verify the effect of heparin on hepcidin expression. It was shown that commercial heparins are potent hepcidin inhibitor *in vitro* in HepG2 cells and *in vivo* in healthy mice and that act by inhibiting the BMP6/SMAD signaling. Heparins are well characterized molecules with some 70 years of clinical experience, and appealing drugs for the treatment of anemia. The major drawback of their strong anticoagulant activity can be overcome. In fact the anticoagulant activity is mostly linked to high binding affinity to antithrombin, which is limited to a specific pentasaccharide, named AT-bs, absent in some heparins, that can be chemically modified (**Figure 5**). The main modifications to reduce or abolish the anticoagulant property are summarized in **Figure 5B** and they are: *N*-desulfation or *N*-acetylation, 2-/6-*O*-desulfation, supersulfation or, more simply, the treatment of heparins by reduction and oxidation, to obtain the so called RO-heparins (Casu et al., 2002). This splits the glycol bonds, increasing molecular flexibility and improving the interaction with targets other than antithrombin. These glycol-split heparins retain various biological functions, including anti-heparanase activity that reduces tumor growth and metastasis in animal models (Ritchie et al., 2011). Some of these compounds are in clinical trials and they have shown little or no toxicity. These glycol-split heparins showed to be potent hepcidin inhibitors *in vitro*, in HepG2 cells and primary hepatocytes, and *in vivo* in mice (Poli et al., 2014). *In vivo* these heparins reduced hepcidin in 6 h with concomitant increase of serum iron and decrease of spleen iron. They inhibited hepcidin also after an acute lipopolysaccharide (LPS) stimulation, and in a mouse model of anemia induced by a single injection of heat-killed *Brucella abortus* (HKBA) these heparins improved the recovery of anemia. The available data indicate that heparins act by sequestering of BMP6 and inhibiting the SMAD1/5/8 signaling. These findings also indirectly suggest a role of liver heparan sulfate proteoglycans (HSPGs) in hepcidin regulation. The main structure of heparin is composed by 70% of *N*-sulfated region (NS, IdoA2SO3- -GlcNSO36SO3), *N*-acetylated region (NA, GlcA-GlcNAc) and mixed NA/NS (GlcA-GlcNSO3; **Figure 5A**). Heparin is structurally analogous to the heparan sulfates (HSs) exposed on the surface of all cells that are known to modulate critical biological events, such as embryonic development, growth regulation and maintenance of normal tissue structure and function (Turnbull et al., 2001). In fact they can act as “receptors” for circulating proteins, including several cytokines and angiogenic growth factors (Casu et al., 2010). Heparin is utilized as a model to study the interaction of molecules with cellular heparan sulfates and to modulate their biological activity (Rusnati et al., 2005). In fact it was recently demonstrated that HSPGs act as coreceptors of BMP2 and BMP4 in facilitating receptor oligomerization (Kuo et al., 2010). The consequences of the BMPs binding to HSPGs vary, depending on the BMP member, cell type targeted and if HSPGs are cell-associated (co-receptor action) or in a free form (antagonist effect). Accordingly, alteration of cell-associated HSPGs by heparinases or by chlorate treatments reduced (Irie et al., 2003) or increased BMP signaling (Jiao et al., 2007). Interestingly, the HS in the liver are highly sulfated, and their inactivation by conditional-KO of key enzymes has effects on lipid homeostasis, but hepcidin has not been analyzed yet (Stanford et al., 2010).

### IL6/STAT3 AXIS

Inflammation induces hepcidin expression mainly through IL6/STAT3 pathway, which can be blocked by anti-IL6 antibody. Siltuximab, an anti-IL6 monoclonal antibody drug used in clinic, was shown to be effective in reducing hepcidin expression in patients with Castleman’s disease (CD) and in improving their anemia (van Rhee et al., 2010). The antibody was used also in patients with renal cell carcinoma and multiple myeloma resulting in a decrease of hepcidin and an increase of hemoglobin (Schipperus et al., 2009; Kurzrock et al., 2013). Tocilizumab is another antibody in clinical use that acts on IL6 activity by binding IL6 receptor. CD patients, treated with it, showed a reduction of serum hepcidin and correction of anemia after 6–12 month treatment (Song et al., 2010). Tocilizumab was used also in monkeys with collagen-induced arthritis with an improvement of anemia (Hashizume et al., 2010). Chemical agents like AG490 that inhibits STAT3 phosphorylation (Caceres-Cortes, 2008) or PpYLKTK that disrupts pSTAT3 dimerization and DNA binding were investigated in cancers with elevated JAK/STAT activity (Zhang et al., 2010). Both compounds decrease IL6-dependent hepcidin expression in differentiated mouse hepatocytes (Fatih et al., 2010) and AG490 also *in vivo* in healthy mice (Zhang et al., 2011).

### ANTI-HEPCIDIN AGENTS

A direct approach is to downregulate hepcidin using RNA interference, taking advantage of the observation that liver is an easy target for siRNAs. This implies the design of RNAi without off-target effects, sufficiently stable *in vivo*, biocompatible and with specific delivery to liver but not to other organs (Wang et al., 2010a). High affinity anti-hepcidin antibodies have been produced and have been engineered to be used *in vivo* and to analyze their effects. They improved the inflammatory anemia in mice induced by HKBA only when co-administrated with erythropoietic stimulating agents (Sasu et al., 2010). Fully humanized mAb against hepcidin (LY2787106) is currently in Phase I for the treatment of cancer-related anemia. Hepcidin blocking proteins were obtained by modifying the lipocalins, natural proteins that bind small hydrophobic ligands and cell surface receptors (Flower, 1996; Schlehuber and Skerra, 2005). They were engineered to produce anticalin PRS-080 that exhibits sub-nanomolar affinity for human hepcidin. Monkeys treated with PRS-080 showed an effective iron mobilization, and studies are in progress on anticalin safety and tolerability *in vivo*. Spiegelmers are synthetic compounds designed to inhibit other molecules. Spiegel means mirror in German and they are mirror-images L-enantiomeric oligonucleotides that bind the targets in a manner similar to antibodies or aptamers. Being nuclease resistant and immunologically passive is suited for *in vivo* application. NOX-H94 is a structured L-oligoribonucleotide, that binds human hepcidin with high affinity, blocking its biological function (Schwoebel et al., 2013). In monkey NOX-H94 prevented the onset of anemia induced by IL6, in human volunteers, it increased indices of iron availability and was safe and well tolerated (Riecke et al., 2012), it also delayed the onset of hypoferremia in volunteers treated with LPS (Van Eijk et al., 2013). The Phase II clinical trials with NOX-H94 are ongoing for patients with anemia of cancer.

### ALTERATION OF HEPCIDIN–FERROPORTIN INTERACTION

Antibodies that block ferroportin binding to hepcidin without affecting its functionality have been described (Leung et al., 2012). They have been engineered and are now in a Phase I trial. A high throughput screening approach discovered a thiol modifier compound (fursultiamine) that prevented ferroportin–hepcidin interaction sequestering the Cys326-HS residue (essential for hepcidin binding, **Figure 3A**) and blocking internalization of ferroportin (Fung and Nemeth, 2013). It could be an interesting agent to be evaluated *in vivo*.

### ERYTHROID FACTORS

Growth differentiation factor 15 (GDF15), is a member of the transforming growth factor-β superfamily. It is produced in erythroid precursor cells and is strongly upregulated in disorders with increased ineffective erythropoiesis, such as β-thalassemia, congenital dyserythropoietic anemias. It was shown to downregulate hepcidin mRNA expression in primary human hepatocytes (Tanno et al., 2007). A synthetic low molecular weight compound (K7174) that enhances GDF15 expression in HepG2 cells was described, and it also reduced hepcidin (Fujiwara et al., 2013). It was claimed a potential therapeutic option to treat ACD. However, this contrasts with the finding that GDF15 deficient mice have normal hepcidin expression and that GDF15 is not required to balance iron homeostasis in response to blood loss (Casanovas et al., 2013). More recently another erythroid factor with strong effect on hepcidin expression was identified, it was named erythroferrone it is stimulated by active erythropoiesis and it suppresses hepcidin expression in hepatic cells (Kautz et al., 2013). It is an important potential target for the control of hepcidin expression.

## ANIMAL MODELS OF INFLAMMATORY ANEMIA

The hepcidin antagonists are expected to find clinical use mainly for the treatment of inflammatory anemia which, although widely diffused in clinical practice, has few animal models with different properties, as recently reviewed (Rivera and Ganz, 2009). Here is a short description of the ones so far described in past and recent papers focusing only on the well-known model.

### LIPOPOLYSACCHARIDE

Lipopolysaccharide injections in the mice induce an inflammatory response, with upregulation of IL6, an increase in Socs3 mRNA, Crp mRNA and hepcidin mRNA and protein and a decrease in serum iron, but generally do not induce anemia (Poli et al., 2014). The activation of hepcidin is fast (4–6 h) and decreases just as quickly. It was shown that anemia could be induced after a single dose of LPS followed a week later by an injection of Zymosan A (a preparation from yeast wall). This, so named ZIGI mouse model, is characterized by high IL6 and hepcidin, increase in spleen iron content with a decrease of liver ferroportin and anemia 5 days after Zymosan injection (Lasocki et al., 2008). The LPS pre-treatment reduces the strong septic shock-like response triggered by Zymosan A which leads to multiple organ dysfunctions (Volman et al., 2005). This interesting model has not been used yet to test the efficacy of hepcidin antagonists.

### TURPENTINE

Turpentine is used to trigger sterile inflammatory response in different animal models. Mice treated with a single subcutaneous injection of turpentine (5 ml/kg) showed induction of hepcidin and hyperferremia (Sakamori et al., 2010). Anemia was described after 3-week of daily treatments, that was accompanied by a reduction of mean corpuscular volume (MCV) and serum iron and a 2–7 fold increase of hepcidin.

### HEAT-KILLED *Brucella* abortus

Heat-Killed *Brucella abortus* agent is the vaccine to prevent Brucellosis in large animals. When injected in mice induce an inflammatory response with anemia. It seems the easiest mouse model of inflammatory anemia, and it was used to verify the activity of hepcidin antagonists like anti-hepcidin antibodies (Sasu et al., 2010) and glycol-split heparins (Poli et al., 2014) to improve anemia *in vivo*. This model of inflammatory anemia has been recently analyzed in depth by the groups of Ganz (Kim et al., 2014) and Rivella (Gardenghi et al., 2014). Both showed that HKBA-treated mice developed a severe anemia, with a nadir after 14 days, followed by a partial recovery after 28 days. They showed hypoferremia and iron-restricted erythropoiesis with normal iron stores, shortened erythrocyte lifespan, and reduced erythropoiesis. The IL6-KO and hepcidin-KO mice showed a milder anemia and a faster recovery confirming the role of inflammation and of hepcidin in the development of anemia in this model. The anemia has multifactorial pathogenesis and hepcidin (that is induced transiently at 6 h after the HKBA injection) appears to play an important role in it. It remains to be evaluated if a model closer to ACD in human can be produced by repeated injections of lower doses of HKBA.

### RAT MODEL OF ANEMIA OF INFLAMMATION

Anemia of inflammation can be obtained in rats with different treatments that have been used for many years. Nowadays a good rat model of ACD is obtained with a single intraperitoneal injection of group A streptococcal peptidoglycan-polysaccharide (PG-APS) with rhamnose. This treatment caused arthritis with involvement of multiple joints (Cromartie et al., 1977). Recently this rat model was analyzed in depth for all iron parameter. After 3 weeks of PG-APS treatment, the rats showed an increase of serum IL6, hepcidin, and ferritin. Spleen ferroportin decreased, resulting in a reduction of iron release from macrophages. These rats develop anemia after 2-week treatment that persists for at least 3 months (Theurl et al., 2009). This anemia has typical features of human ACD (mild to moderate normocytic and normochromic anemia) with inflammation. This rat model was used to test the effect of different hepcidin inhibitors with positive results (Theurl et al., 2011).

#### Modified adenine-induced kidney disease rat model

Another interesting rat model that develops CKD is obtained in rodent with a 0.75% adenine diet (modified adenine) for 3 weeks followed by a control diet for 5 weeks. This protocol improved survival (90%) maintaining persistent kidney disease and more severe anemia (Sun et al., 2013). This model was used to evaluated the effect of the BMP inhibitor LDN-193189 (Sun et al., 2013). Adenine-treated rats showed increased liver hepcidin mRNA, decreased serum iron, increased spleen iron content, low hemoglobin, and low erythropoietin levels. LDN-193189 treatment reduced hepatic hepcidin mRNA, mobilized stored iron and increased hemoglobin content of reticulocytes.

### GENETIC MODEL OF ANEMIA

Iron refractory iron deficiency anemia is an autosomal recessive human disorder characterized by congenital hypochromic, microcytic anemia, very low mean corpuscular erythrocyte volume, low transferrin saturation, poor response to oral iron supplementation and partial response to parenteral iron therapy. The mouse models that mimic this disorder were obtained by two groups (Du et al., 2008; Folgueras et al., 2008). One was produced by chemically induced mutation that causes splicing defect in the transmembrane serine protease in gene *Tmprss6*. The other one is the KO model produced by a duplication of an entire region of *Tmprss6* gene. In the two models matriptase-2 is inactivated and is characterized by progressive loss of body but not facial hair (“*mask* phenotype”) and microcytic anemia. The *mask* phenotype results from reduced absorption of dietary iron and iron retention in duodenal enterocytes, low ferroportin, and iron deficiency anemia caused by high levels of hepcidin (Folgueras et al., 2008). *Mask* homozygotes are slightly smaller than their heterozygous littermates, and adult female homozygotes are infertile whereas male homozygotes retain fertility. This mouse with a direct implication of hepcidin upregulation is probably the best model to study the long term effects of hepcidin inhibitors with the aim at solving anemia.

## CONCLUSION

This review shows that many laboratories are studying different pharmacological means to neutralize hepcidin expression or activity in order to cure inflammatory anemia. They produced a number of promising approaches, and some of them have been tested in animal models. Most of them seemed to be effective in reducing hepcidin expression or activity under acute conditions, but it is still unclear if and how they are efficient in the treatment of anemia. One of the problems is the lack of adequate animal models for inflammatory anemia, as indicated above. Mice models are rather complex, and rat models seems to mimic more closely the human disease, but the absence of transgenic rats for hepcidin and inflammatory cytokines does not allow a detailed characterization. Monkeys have been used to induce inflammatory response, but not anemia (Cooke et al., 2013). The described antagonists (**Table 1**) originate from different and novel biotechnological techniques, including humanized anti-hepcidin antibodies, aptamers, anticalin, siRNAs, and the old traditional heparin. Some of them are in clinical trials, and perhaps in a few years we will know if the downregulation of hepcidin really meets the expectation to improve the anemia in most, or some chronic diseases. All the antagonists have some advantages and problems. For example the humanized antibodies have a long half-life *in vivo*, but their production is highly expensive. Aptamer and anticalin inactivate hepcidin, but their fate is unclear. The specific and efficient delivery of siRNA is complex. Non-anticoagulant heparins are probably the best known, most convenient and safer agents. After 70 or more years of use in clinic, most of problems and side effects of heparins are known. They include thrombocytopenia, elevation in serum aminotransferase, hyperkalemia, alopecia and osteoporosis, but they occur rarely and are transient. The removal of anticoagulant activity has been resolved and the clinical trials of these agents as hepcidin antagonist should not be far away. Also the negative effects of treatments for hepcidin inhibition should be taken into account. They are expected to increase systemic iron availability and absorption, which may favor iron-dependent oxidative damage in some parenchymal tissues. If the pharmacological agents are capable to cross BBB and enter the brain, they may alter CNS iron homeostasis with unpredictable effects. In addition, hepcidin is known to have an antimicrobial activity, which is considered low, but its biological role needs to be established.

**Table 1:** Hepcidin inhibitors and corresponding targets.

Inhibitors	Target	Reference
**BMPs/BMPr complex**
sHJV-Fc	Inhibitors of BMPs/SMAD pathway	Babitt et al. (2007), Andriopoulos et al. (2009), Theurl et al. (2011), Wang et al. (2012)
LDN-193189	Inhibitor of phosphorylation of BMPs receptor type I	Cuny et al. (2008), Steinbicker et al. (2011), Theurl et al. (2011), Wang et al. (2012), Sun et al. (2013), Saeed et al. (2012)
siHJV, siTfR2	Degradation of HJV or TfR2 mRNA	Akinc et al. (2011)
Anti-BMP6 antibody	Sequestration of BMP6	Andriopoulos et al. (2009), Corradini et al. (2010), Wang et al. (2012)
Heparin	Inhibitors of BMPs/SMAD pathway	Poli et al. (2011, 2014)
**IL6/STAT3 axis**
Anti-IL6r (Tocilizumab)	Sequestration of IL6 receptor	Song et al. (2010), Hashizume et al. (2010)
Anti-IL6 (Siltuximab)	Sequestration of IL6	Schipperus et al. (2009), van Rhee et al. (2010), Kurzrock et al. (2013)
AG490	Inhibitor of STA3 phosphorylation	Fatih et al. (2010), Zhang et al. (2011)
PpYLKTK	Disruptor of STAT3 dimerization	Fatih et al. (2010)
**Anti-hepcidin agents**
siHep	Degradation of hepcidin mRNA	Pharmaceuticals, A. ALN-HPN: refractory anemia 2011; Xenon. Isis and Xenon collaborate to develop antisense drugs against hemojuvelin and hepcidin 2010.
Anti-hepcidin antibody	Sequestration of hepcidin protein	Sasu et al. (2010)
Anticalin	Sequestration of hepcidin protein	Congress of the International BioIron Society (BioIron 2011) Vancouver, Canada. American Journal of Hematology 2011; 86:E48.
Spiegelmers	Sequestration of hepcidin protein	Schwoebel et al. (2013), Riecke et al. (2012), Van Eijk et al. (2013)
**Alteration of hepcidin–ferroportin interaction**
anti-ferroportin antibodies	Interfering with hepcidin binding to ferroportin	Leung et al. (2012)
Fursultiamine	“Sequestration” of Cys326-HS on FPN heparin binding site	Fung et al. (2013)

## Conflict of Interest Statement

The authors declare that the research was conducted in the absence of any commercial or financial relationships that could be construed as a potential conflict of interest.

## ABBREVIATIONS

BMP: bone morphogenetic protein
SMAD: sons of mothers against decapentaplegic
STAT: signal transducer and activator of transcription
IL-6: interleukin-6
LPS: lipopolysaccharides
GAG: glycosaminoglycan.
